# Epidemiological and Evolutionary Analysis of Dengue-1 Virus Detected in Guangdong during 2014: Recycling of Old and Formation of New Lineages

**DOI:** 10.4269/ajtmh.18-0951

**Published:** 2019-08-05

**Authors:** Jianhai Yu, Xujuan Li, Xiaoen He, Xuling Liu, Zhicheng Zhong, Qian Xie, Li Zhu, Fengyun Jia, Yingxue Mao, Zongqiu Chen, Ying Wen, Danjuan Ma, Linzhong Yu, Bao Zhang, Wei Zhao, Weiwei Xiao

**Affiliations:** 1 Guangdong Provincial Key Laboratory of Tropical Disease Research, School of Public Health, Southern Medical University, Guangzhou, China;; 2 Guangdong Women and Children’s Hospital, Guangzhou Medical University, Guangzhou, China;; 3 Department of Traditional Chinese Medicine, Southern Medical University, Guangzhou, China;; 4 Guangzhou Key Laboratory of Drug Research for Emerging Virus Prevention and Treatment, School of Pharmacy, Southern Medical University, Guangzhou, China;; 5 School of Public Health, Guangdong Medical University, Dongguan, China

## Abstract

The incidence of dengue is increasing in Guangdong, China, with the largest outbreak to date in 2014. Widespread awareness of epidemiological and molecular characteristics of the dengue virus (DENV) is required. In 2014, we isolated the virus from patients and sequenced its genome. The sequences of DENV isolated from Guangdong and other countries screened since 2005 were studied to establish molecular evolutionary databases along with epidemiological data to explore its epidemiological, phylogenetic, and molecular characteristics. Causes underlying the occurrence of the dengue epidemic included importation and localization of the virus. The number of indigenous cases significantly exceeded that of imported cases. Dengue virus 1 is the most important serotype and caused the long-term epidemic locally. Based on the data available since 2005, DENV1 was divided into three genotypes (I, IV, and V). Only genotypes I and V were detected in 2014. In 2014, an epidemic involving old lineages of DENV1 genotype V occurred after 2 years of silence. The genotype was previously detected from 2009 to 2011. Genotype I, which caused recent epidemics, demonstrated a continuation of new lineages, and a predictive pattern of molecular evolution since 2005 among the four lineages was present. The DENV isolated from Guangdong was closely related to those causing large-scale epidemics in neighboring countries, suggesting the possibility of its import from these countries. The lack of sufficient epidemiological data and evidence on the local mosquito-borne DENV emphasizes the importance of studying the molecular evolutionary features and establishing a well-established phylogenetic tree for dengue prevention and control in Guangdong.

## INTRODUCTION

Dengue virus (DENV), a mosquito-borne flavivirus, is transmitted primarily by *Aedes aegypti* and *Aedes albopictus*, causing an acute infectious disease named dengue fever (DF),^1^ which gives rise to public health problems in tropical and subtropical regions worldwide, such as China, Singapore, and Brazil.^2–4^ With the recent revision of the WHO dengue classification scheme, dengue patients are classified as having either dengue or severe dengue. The former refers to patients who recover without major complications, whereas the latter points to those who have any of the following conditions: plasma leakage resulting in shock, accumulation of serosal fluid sufficient to cause respiratory distress, or both; severe bleeding; and severe organ impairment.^5^ Before 1970, only nine countries had experienced severe dengue epidemics, but the disease is now endemic in more than 100 countries.^6^ In recent decades, the spreading disease causes rapid upsurge in morbidity. One recent estimate indicates 390 million dengue infections per year, of which 96 million manifest clinically.^7,8^ Hence, wasting a lot of health resources and causing the growing global burden of disease, DF is regarded as the most widely distributed vector-borne disease with the highest morbidity and great harm.^5^

The genome of DENV is a single-stranded, positive-sense RNA, and its single open reading frame encodes a polyprotein consisting of three structural proteins, which are as follows: the capsid, membrane-associated, and envelope (E) proteins. In addition, seven nonstructural proteins, NS1, NS2A, NS2B, NS3, NS4A, NS4B, and NS5, are also present in its structure.^9^ Four distinct DENV serotypes (DENV1, DENV2, DENV3, and DENV4) have been identified, and the extensive diversity within DENV1 enables it to recognize different genotypes, such as genotype I (Southeast Asia and East Africa), genotype II (Thailand), genotype III (Malaysia), genotype IV (South Pacific), and genotype V (America/Africa).^10^ Dengue genotypes are phylogenetically distinct clusters of viruses, often associated with specific geographical regions, that are linked to epidemics of varying intensities and disease severity.^11,12^ Phylogeny is a science that makes use of a set of relationships among groups of genes or organisms and reflects their evolutionary history. The maximum likelihood method is used to describe and analyze biological sequences.^13^ Alignment of the nucleic acid sequences of the DENV followed by its phylogenetic analysis and subsequent generation of phylogenetic trees revealed information regarding its genetic evolution and epidemiology of the disease worldwide.^14–16^ The E protein of the DENV is responsible for its tropism and virulence.^17^ The gene encoding the E protein has demonstrated its usefulness for decades for the phylogenetic reconstruction of the DENV. Thus, complete analysis of the coding region can help assign the correct DENV genotype and infer the relationships within genotypes and lineages accurately.^18^ Therefore, the E protein provides adequate resolution to characterize genetic relationship and evolution of the DENV.^19^

Guangdong Province is located in the southern mainland of China and experiences tropical and subtropical monsoon climates with a hot and rainy environment that supports breeding of mosquitos, leading to epidemics and very high incidences of DF.^20,21^ The first outbreak of dengue in the Foshan city of Guangdong Province occurred in 1978, after which periodic infections and transmission of all four serotypes of dengue have occurred in the past 30 years.^22^ The DENV found in these areas may have different origins. There were two DENV2 outbreaks in Foshan in 1993 and 1998, respectively. Phylogenetic analysis revealed that isolates from the 1998 epidemic and DENV4 from the 1978 epidemic and the 1990 epidemic were closely related to those from the 1993 epidemic in Thailand, 1973 epidemic in Indonesia, and the 1984 epidemic in the Philippines, respectively. Since 1990, however, DENV1 has been mainly isolated from the infected cases, and its continued existence in Guangdong Province indicated that endemic infectious agents of dengue may be circulating locally. Sequence analysis of the viruses causing the epidemics at different time points revealed that the isolates were closely related to each other, implying that DENV1 had probably circulated locally and caused the epidemics.^23^ Since 2009, all four serotypes were derived from autonomous patients from different outbreak localities in Guangdong Province.^24^ In 2014, a total of 45,236 cases of dengue were reported, which exceeded the total number of cases reported over the previous 10 years. Although three serotypes of the DENV (DENV1, DENV2, and DENV3) were identified, DENV1 was found to be the major causative agent responsible for 98.72% of all 21,928 laboratory-confirmed cases diagnosed during this outbreak.^25^ A recent study revealed that the detected sequences belonged to viruses of multiple origins, but the strain isolated in 2014 possibly originated from the isolates of 2013.^26^ It can be reasonably speculated that the infectious agents of DENV1 from the endemic, which were circulating locally, played a crucial role in causing the dengue epidemic in Guangdong Province. However, the data mentioned previously are from the studies based on the outbreaks of the particular year and have the limitation of space and time. As a result, comprehensive evaluation of the epidemiological situation and molecular evolution of the viral agents is of significance to warn against their risks and establish preventive and control measures for DF.

Based on DENV1 isolated from the outbreak in 2014, we systematically collected the E protein gene from 2005 to 2018 from GenBank. With the epidemiological data since 2005 supplied by the Guangdong Provincial CDC, we studied phylogenetics, molecular characteristics, and epidemiology to strengthen the foundational research of DENV1 for the prevention of large-scale dengue epidemics, providing preventive and control measures of DF with important evidence.

## MATERIALS AND METHODS

### Ethics statement.

As this research involved human blood, the aims of our study were explained to all the dengue patients involved (all were adults) and all provided written informed consent. The collection methods of clinical samples and epidemiological data were reviewed and approved by the Institutional Ethics Review Board of Southern Medical University and were carried out in accordance with the approved guidelines. Samples were selected randomly based on the laboratory diagnosis and clinical signs.

### Sample collection and epidemiological data.

Dengue virus RNA samples (*n* = 29) were obtained from the Guangdong Provincial Maternal and Child Care Service. All samples were extracted from patients suspected of dengue and were confirmed by reverse transcription-polymerase chain reaction (RT-PCR) using specific primers. The steps included an initial denaturation (94°C, 2 minutes); 35 cycles of denaturation (94°C, 30 seconds), annealing (60°C, 30 seconds), and extension (72°C, 1 minute); and a final extension (72°C, 10 minutes) (Table 1). Positive samples were stored at –80°C. Another two serum samples from individuals suspected of having dengue were provided by the Health Care Center of Guangdong International Travel. Twenty-five DENV strains were isolated by the Guangdong CDC on C6/36 cells. The strains obtained were amplified on C6/36 cells maintained in RPMI-1640 supplemented with 2% fetal bovine serum at 37°C in an atmosphere of 5% CO_2_. After complete cytopathic effects were observed, the culture supernatants were collected.

**Table 1:** Primers used for identifying the serotype of dengue virus (DENV) strains and amplifying and sequencing the envelope (E) gene of DENV1 in this study

ID primer	Primer sense F (5′–3′)	Primer antisense R (5′–3′)	Amplicon (bp)
Type-specific primers			
DENV1	TCAATATGCTGAAACGCGCGAGAAACCG	CGTCTCAGTGATCCGGGGG	482
DENV2	TCAATATGCTGAAACGCGCGAGAAACCG	CGCCACAAGGGCCATGAACAG	119
DENV3	TCAATATGCTGAAACGCGCGAGAAACCG	TAACATCATCATGAGACAGAGC	290
DENV4	TCAATATGCTGAAACGCGCGAGAAACCG	CTCTGTTGTCTTAAACAAGAGA	392
E gene			
1	TAGCACATGCCATAGGAA	CTGGGTCTCAGCCACTTC	1,028
2	ATGCAAAGAAGCAGGAAG	AATTTGTATTGCTCTGTCCA	856

The GenBank accession number KM204119.1 was used for DENV1, whereas KM204118.1 was used for DENV2, KU050695.1 for DENV3, and AY947539.1 for DENV4.

Epidemiological data on all samples were collected from the supplier. The information included collection dates, collection regions, clinical manifestations, and travel histories. Epidemiological data on DF since 2005 were supplied by the Guangdong Provincial CDC, including the number of cases (corresponding with each city), the distribution of imported cases and indigenous cases, DENV serotype data confirmed by laboratory, and other information. We carried out statistical analysis. The picture of the administrative divisions of Guangdong Province used for the heat map was obtained from the Wikimedia Commons open-source map site (https://commons.wikimedia.org/wiki/File:Heyuan_map2005.jpg) with free use of the information permitted.

### Viral genome amplification and sequencing.

Viral RNA was extracted from 140 μL of serum sample or culture supernatant using the QIAamp^®^ Viral RNA Mini Kit (Qiagen, Hilden, Germany). A PrimeScript^™^ II First Strand cDNA Synthesis Kit (TaKaRa Bio, Shiga, Japan) was used for the transcription of DENV RNA. Type-specific primers (Table 1) were used to confirm the serotype through RT-PCR using the LightCycler480^®^ instrument (Roche Diagnostics, Roche Instrument Center AG, Rotkreuz, Switzerland). Targeted fragments of approximately 856 bp and 1,028 bp were used to amplify E gene cDNA using two pairs of primers (Table 1). After DNA sequencing, two targeted fragments with overlapping sequences were spliced into a complete E gene sequence. The amplification process for the E gene involved an initial denaturation (94°C, 2 minutes); 40 cycles of denaturation (94°C, 30 seconds), annealing (58°C, 30 seconds), and extension (72°C, 1 minute); and a final extension (72°C, 10 minutes). After sequencing performed by Sangon Biotech (Shanghai, China), data were uploaded to the GenBank database.

### Genome and phylogenetic analysis.

The sequences were saved by DNASTAR (http://www.dnastar.com/)^27^ and were identified by BLAST (http://blast.ncbi.nlm.nih.gov/Blast.cgi).^28^ The sequence alignment was conducted in BioEdit (http://www.mbio.ncsu.edu/bioedit/bioedit.htmL)^29^ with CLUSTALW2 (http://www.ebi.ac.uk/Tools/msa/clustalw2/).^29^ Phylogenetic analysis was performed by using MEGA6.0 (http://www.megasoftware.net/)^30^ through the maximum-likelihood method. The Tamura–Nei model and Gamma distribution with a bootstrap of 1,000 replications were used for the maximum-likelihood method tree based on the analysis of the best-fit model for each dataset, as provided by the software.

Two phylogenetic trees for the E gene and one for the complete genome of DENV1 were established. The main focus was the analysis of the molecular characterization during the dengue outbreak in Guangdong, China, in 2014. For this, contemporaneous E gene sequences in other countries were added to make a comprehensive evaluation of the epidemiological situation and molecular evolution in the large 2014 dengue outbreak in Guangdong. At the same time, E gene sequences of DENV1 since 2005, comprising 187 sequences from Guangdong and 1,886 from other countries, were downloaded from GenBank. After excluding several sequences with uncertain epidemiological data, representative epidemic strains in every lineage were screened using phylogenetic methods. Since then, a molecular evolution database for the DENV1 E gene in Guangdong and other countries has been established. Based on representative strains of the E gene in lineages of the 2014 outbreak, as well as the molecular evolution database, we analyzed molecular characterization and possibility of local circulation for DENV1 since 2005 in Guangdong.

### Three-dimensional (3D) structure prediction of DENV1 protein E and molecular docking with 78-kDa glucose-regulated protein (GRP78).

Amino acid (AA) sequences of DENV1 protein E were translated by using DNASTAR. Protein 3D structures were simulated by Discovery Studio 2.5 (DS2.5) (http://accelrys.com/products/collaborative-science/biovia-discovery-studio/)^31^ through the homology modeling method based on the MODELER program. The optimal protein 3D model was selected by combining the Probability density function and discrete optimized protein energy, and the reliability of the model was evaluated using the Ramachandran plot and Profile-3D. The optimal protein structure model was used to perform protein docking calculations with the GRP78 protein (PDB number: 3LDP) using the ZDOCK algorithm in DS2.5, and RDOCK was used to further optimize the docking configuration to minimize energy. Finally, we analyzed AA sites in the binding interface of the optimal docking model.

## RESULTS

### Epidemiological findings.

**Figure 1.**
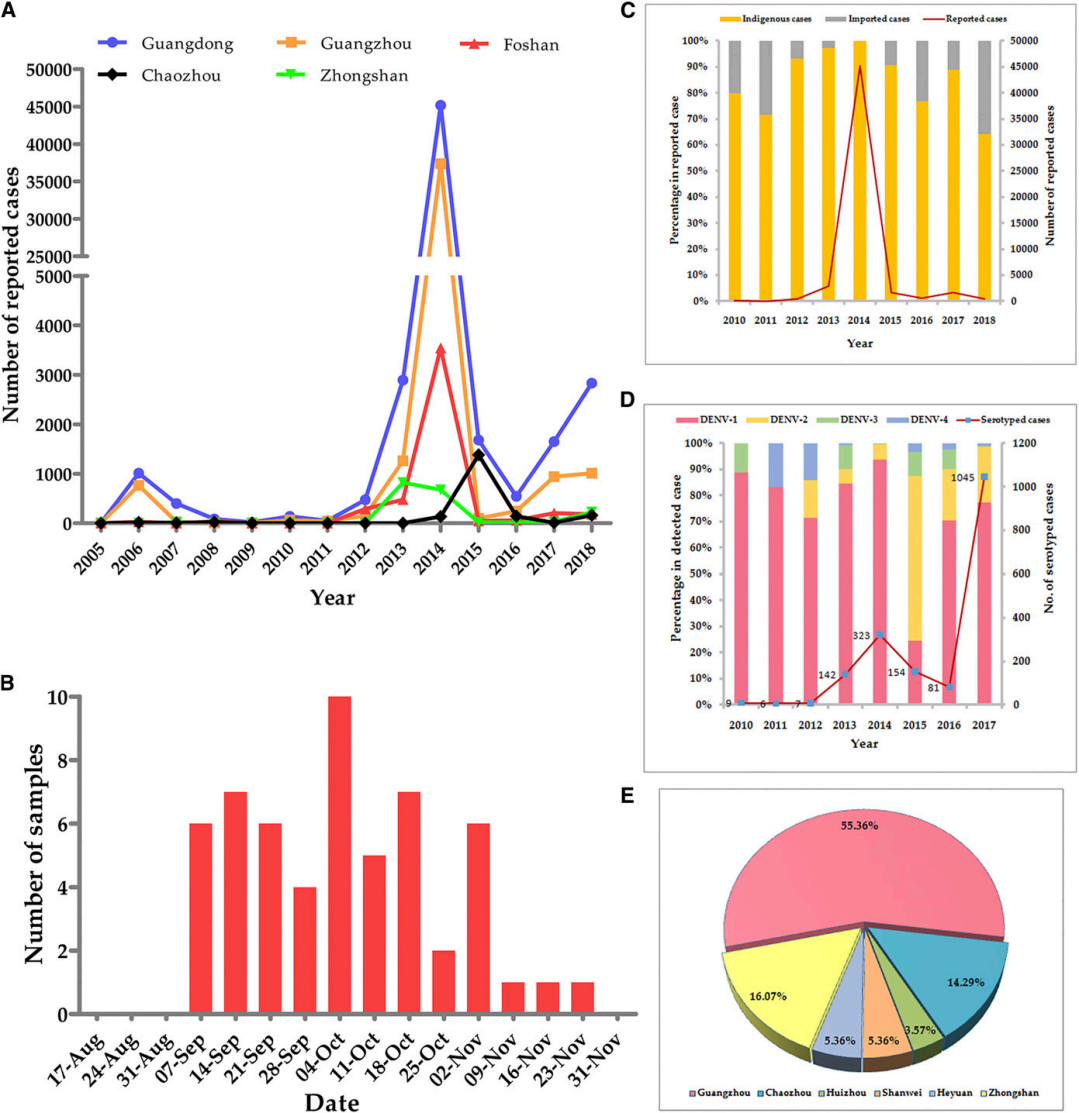
Epidemiological data of dengue outbreaks in Guangdong since 2005 and sample information collected in 2014. (**A**) The number of reported cases from Guangdong and the four major centers of epidemics as of November 4, 2018. (**B**) Time distribution of samples collected in 2014. (**C**) Percentage of indigenous and imported cases reported annually. (**D**) Identification of dengue virus serotypes annually. (**E**) Regional distribution of samples collected in 2014. This figure appears in color at www.ajtmh.org.

**Figure 2.**
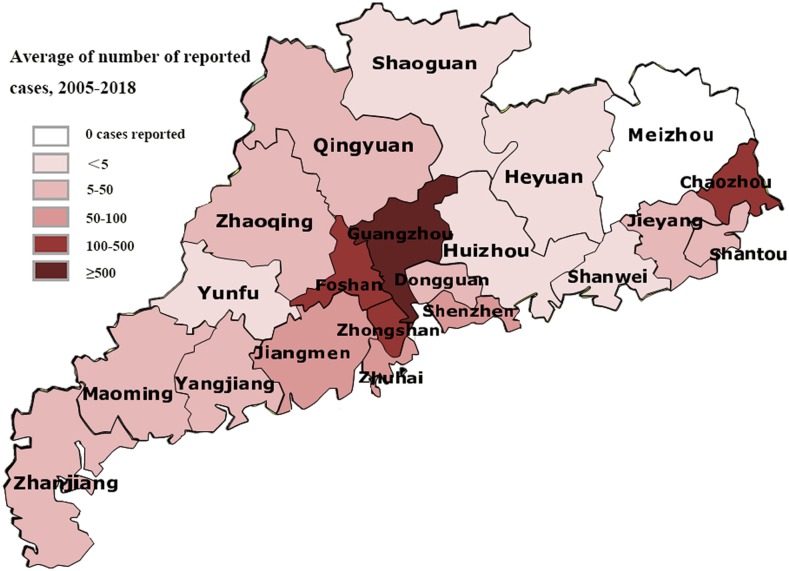
Average annual number of dengue cases reported by parish since 2005–2018 in Guangdong, China (data collected until November 4, 2018). This figure appears in color at www.ajtmh.org.

Since 2005, epidemics of dengue in Guangdong have been characterized by periodic outbreaks, the coexistence of importation and localization, and the significantly increased number of indigenous cases compared with that of imported cases (Figure 1A and C). The incidence peaked in 2006 and 2014. Especially, during the large outbreak in 2014, a total of 45,236 cases of dengue were reported. In 2017, 1,650 cases were reported, with a continuous rising trend in comparison to 544 cases in 2016 (Figure 1A). More seriously, as of November 4, 2018, a total of 2,835 cases were reported, an increase of 107.1% over the same period in 2017. Among them, 257 cases were imported, which was 50.3% higher than those in the same period in 2017; 2,578 cases were local cases, which was 115.2% higher than those in the same period in 2017. Based on the map of Guangdong Province, we diagrammed the distribution of the cumulative number of cases since 2005. This showed that Guangzhou, Foshan, Zhongshan, and Chaozhou were the main epidemic areas. The trends of the epidemics were anastomotic, with periods of fluctuation in Guangdong Province (Figure 2). After 2010, the epidemic trend featured the gradual coexistence of various serotypes, with up to four serotypes emerging in recent years. Each DENV serotype was identified. Dengue virus 1 was the major cause of the outbreak of 2014, and it continued to be detected in Guangdong Province as the primary serotype, except in 2015. It is worth noting that the proportion of dengue 2 virus in the total cases also gradually increased (Figure 1D).

Data of 56 samples were used to analyze the overall epidemiology. All patients who provided the samples had mild dengue and had no travel history (Table 2). Generally, the collection date distribution was concentrated in September and October (Figure 1B). The endemic areas were primarily located in Guangzhou, followed by Zhongshan and Chaozhou, accounting for 85.72% of all samples. Heyuan, Huizhou, and Shanwei were included in the collection areas (Figure 1E). Dengue virus 1 was the major causative agent of this outbreak. The serotype detected through type-specific primers (Table 1) was DENV1, which is similar to the results from Guangdong CDC that of 323 DF cases involved, 303 cases were due to DENV1 infection (Figure 1D, Table 2).

**Table 2:** Epidemiological data of DENV-1 samples used in this study for partial (envelope gene; *n* = 51) and complete (*n* = 5) sequencing of the genome

ID of strain	Origin of strain	Genes sequenced	City of collection	Collection date	GenBank accession
P1003	Serum	Envelope	Guangzhou	October 2, 2014	MG560209
P1005	Serum	Envelope	Guangzhou	October 3, 2014	MG560210
P1007	Serum	Envelope	Guangzhou	October 3, 2014	MG560211
P1023	Serum	Envelope	Guangzhou	October 16, 2014	MG560212
P1025	Serum	Envelope	Guangzhou	October 18, 2014	MG560213
P1027	Serum	Envelope	Guangzhou	October 18, 2014	MG560214
P1029	Serum	Envelope	Guangzhou	October 26, 2014	MG560215
P1035	Serum	Envelope	Guangzhou	October 26, 2014	MG560216
P1036	Serum	Envelope	Guangzhou	October 8, 2014	MG560217
P1040	Serum	Envelope	Guangzhou	October 8, 2014	MG560218
P1046	Serum	Envelope	Guangzhou	October 28, 2014	MG560219
P1047	Serum	Envelope	Guangzhou	September 2, 2014	MG560220
P1052	Serum	Envelope	Guangzhou	September 6, 2014	MG560221
P1056	Serum	Envelope	Guangzhou	September 12, 2014	MG560222
P1057	Serum	Envelope	Guangzhou	September 12, 2014	MG560223
P1058	Serum	Envelope	Guangzhou	September 3, 2014	MG560224
P1060	Serum	Envelope	Guangzhou	September 15, 2014	MG560225
P1066	Serum	Envelope	Guangzhou	September 16, 2014	MG560226
P1070	Serum	Envelope	Guangzhou	September 20, 2014	MG560227
P1074	Serum	Envelope	Guangzhou	September 13, 2014	MG560228
P1075	Serum	Envelope	Guangzhou	September 25, 2014	MG560229
P1078	Serum	Envelope	Guangzhou	September 25, 2014	MG560230
P1081	Serum	Envelope	Guangzhou	September 29, 2014	MG560231
P1083	Serum	Envelope	Guangzhou	September 29, 2014	MG560232
P1085	Serum	Envelope	Guangzhou	September 20, 2014	MG560233
P1086	Serum	Envelope	Guangzhou	September 27, 2014	MG560234
P1089	Serum	Envelope	Guangzhou	November 1, 2014	MG560235
P1094	Serum	Envelope	Guangzhou	November 2, 2014	MG560236
P1098	Serum	Envelope	Guangzhou	November 5, 2014	MG560237
P1233	Isolated (C6/36)	Envelope	Chaozhou	October 8, 2014	MG560239
P1251	Isolated (C6/36)	Envelope	Chaozhou	October 16, 2014	MG560240
P1252	Isolated (C6/36)	Envelope	Chaozhou	October 16, 2014	MG560241
P1253	Isolated (C6/36)	Complete	Chaozhou	October 16, 2014	MG560269
P1254	Isolated (C6/36)	Envelope	Chaozhou	October 17, 2014	MG560243
P1257	Isolated (C6/36)	Complete	Chaozhou	October 20, 2014	MG560268
P1258	Isolated (C6/36)	Complete	Chaozhou	October 20, 2014	MG560267
P1260	Isolated (C6/36)	Envelope	Chaozhou	October 26, 2014	MG560246
P1263	Isolated (C6/36)	Envelope	Zhongshan	September 1, 2014	MG560247
P1264	Isolated (C6/36)	Envelope	Zhongshan	September 1, 2014	MG560248
P1265	Isolated (C6/36)	Envelope	Zhongshan	September 5, 2014	MG560249
P1275	Isolated (C6/36)	Envelope	Zhongshan	September 10, 2014	MG560250
P1276	Isolated (C6/36)	Envelope	Zhongshan	September 10, 2014	MG560251
P1277	Isolated (C6/36)	Envelope	Zhongshan	September 11, 2014	MG560252
P1278	Isolated (C6/36)	Complete	Zhongshan	September 11, 2014	MG560266
P1296	Isolated (C6/36)	Envelope	Zhongshan	September 17, 2014	MG560254
P1297	Isolated (C6/36)	Envelope	Zhongshan	September 17, 2014	MG560255
P1301	Isolated (C6/36)	Envelope	Shanwei	October 2, 2014	MG560256
P1306	Isolated (C6/36)	Envelope	Shanwei	October 4, 2014	MG560257
P1307	Isolated (C6/36)	Envelope	Shanwei	October 4, 2014	MG560258
P1340	Isolated (C6/36)	Envelope	Huizhou	November 14, 2014	MG560259
P1341	Isolated (C6/36)	Envelope	Huizhou	November 18, 2014	MG560260
P1344	Isolated (C6/36)	Envelope	Heyuan	September 29, 2014	MG560261
P1346	Isolated (C6/36)	Complete	Heyuan	October 1, 2014	MG560265
P1351	Isolated (C6/36)	Envelope	Heyuan	October 8, 2014	MG560263
P120c-1	Serum	Envelope	Guangzhou	October 6, 2014	MG560238
P240c-1	Serum	Envelope	Guangzhou	September 28, 2014	MG560264

All patients had mild dengue and had no travel history.

### Phylogenetic analysis of DENV1 outbreak in 2014.

**Figure 3.**
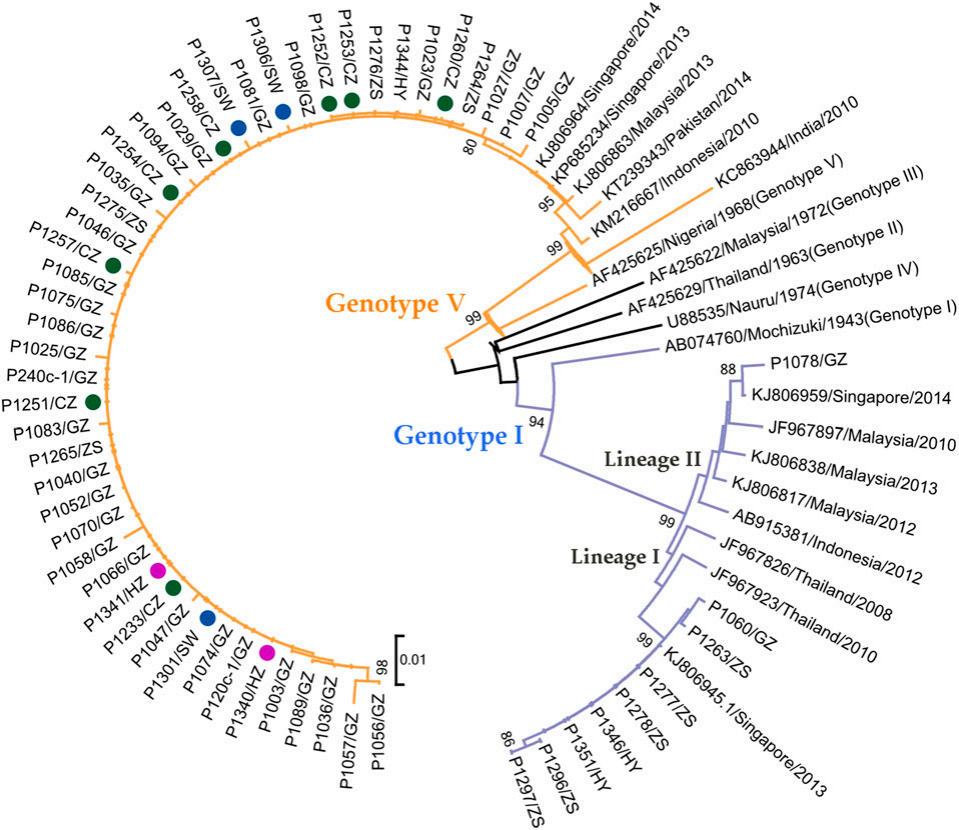
Phylogenetic analysis of DENV1 detected from the 2014 outbreak in Guangdong. Fifty-six envelope gene sequences were isolated in our study. These and 19 reference sequences from GenBank were used to construct the phylogenetic tree. Asian genotype I and American/African genotype V were involved in this outbreak. Forty-seven sequences identified as genotype V clustered into the same clade, whereas genotype I was divided into Lineages I and II. Thirteen sequences from Shanwei (blue circles), Chaozhou (green circles), and Huizhou (violet circles) all belonged to genotype V. This figure appears in color at www.ajtmh.org.

All 56 E genes were sequenced to construct phylogenetic trees. The dengue outbreak in 2014 involved Asian genotype I and American/African genotype V. Phylogenetic analysis showed that most of the sequences were clustered into a unified clade whose distribution differed in each city. Among them, 47 E gene sequences were identified as genotype V with 98.9–100% similarity. They clustered into the same clade, which was closely related to the DENV1 sequences in Malaysia, Singapore, and India and evolved into a lineage of genotype V. During 2013–2014, this lineage was involved in a co-epidemic in Guangdong Province, Malaysia, and Pakistan, with a continuous high-level local prevalence especially in Singapore. Significantly, this lineage was detected in six cities in our collection; 13 sequences from Shanwei, Chaozhou, and Huizhou all belonged to genotype V. In addition, another nine sequences of the E gene were identified as genotype I, with two different lineages. Lineage I involved eight sequences in our collection and other sequences in Singapore and Thailand in recent years, with a 99.6–100% similarity. Only one sequence in Guangzhou contributed to Lineage II, which had been involved in local epidemics in Malaysia, Singapore, and Indonesia for many years (Figure 3).

### Phylogenetic analysis of Guangdong since 2005.

We downloaded E gene sequences since 2005, comprising 187 from Guangdong and 1886 from other countries. Databases of E gene molecular evolution were created via a screening process based on epidemiologic and phylogenetic methods. Among them, 81 strains in Guangdong constituted the local database, whereas 403 strains in other countries made up the imported database.

**Figure 4.**
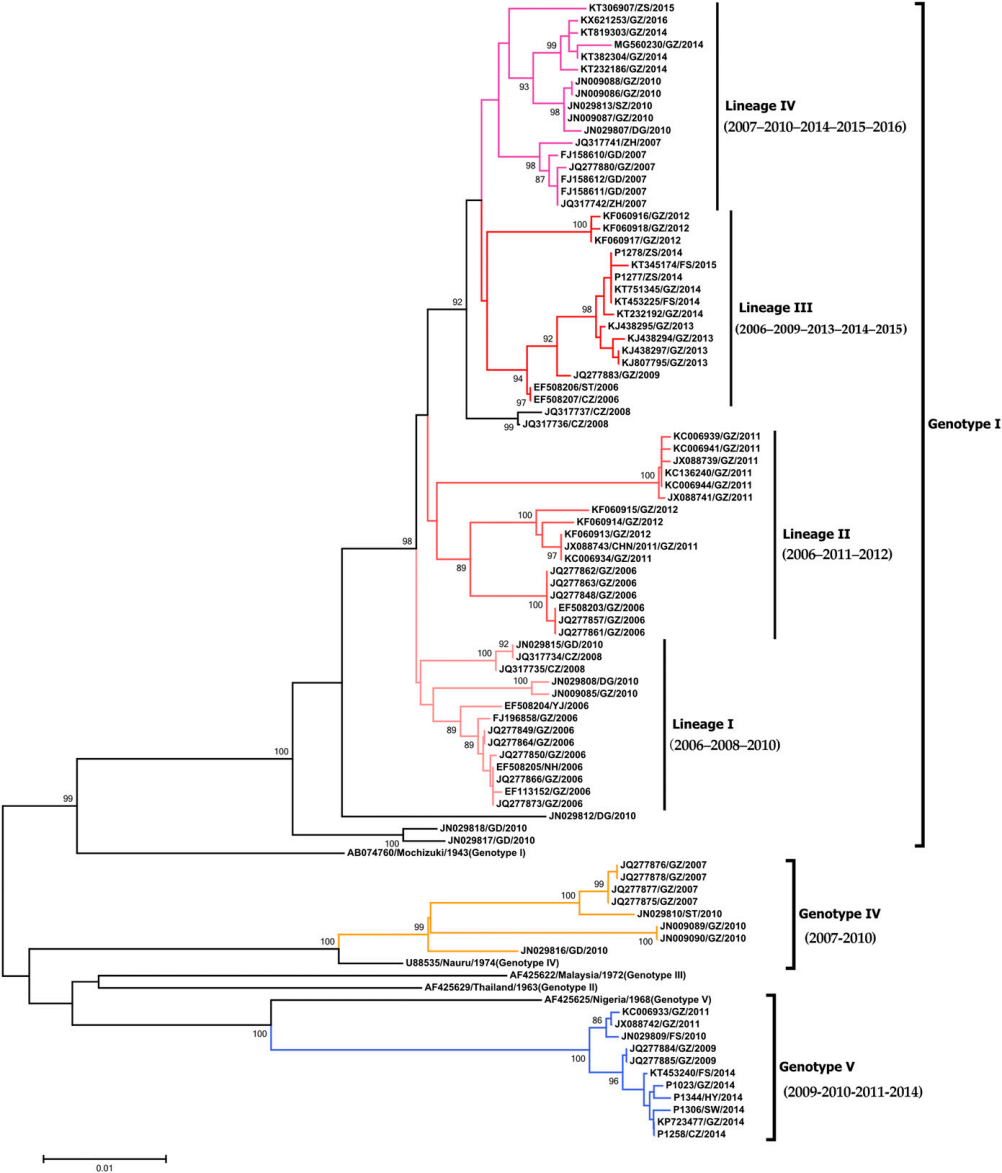
Phylogenetic tree of a local molecular evolution database constructed by the envelope (E) gene in Guangdong since 2005. Genotypes I, IV, and V were detected in 88 E genes of DENV1 strains covering every epidemic year. Genotype I extensively circulated in Guangdong and covered every epidemic year. The genotype branched into four molecular evolution lineages with temporal continuance. Genotype IV was only detected in 2007 and 2010. Genotype V circulated from 2009 to 2011 and then, after a 2-year silence, reappeared in 2014. The colored sequence represents DENV1 in Guangdong Province. The same color in genotype I represents the viral sequences cluster into the same lineage. Yellow and blue represent genotypes IV and V, respectively. This figure appears in color at www.ajtmh.org.

Based on the evolutionary lineage of DENV1 detected in 2014 in Guangdong, seven sequences (P1023, P1078, P1258, P1277, P1278, P1306, and P1344) were added to the local database for phylogenetic evolution analysis. Distribution of the collection date of DENV1 strains used for analysis was highly consistent with the dengue epidemic, with a low prevalence in 2005, 2008, 2009, and 2011. Genotypes I, IV, and V were detected since the 2005 dengue epidemic. Genotype I, which had extensively circulated in Guangdong and was detected in every year of an epidemic, was branched into four molecular evolutionary lineages. During the outbreak in 2006, three lineages were first formed as the basis of circulation. Over the next decade, sequences in other prevalent years were included into these branches in close relationship, with 2006–2008–2010 for Lineage I, 2006–2011–2012 for Lineage II, and 2006–2009–2013–2014–2015 for Lineage III. Sequences in 2007 formed a branch named Lineage IV for 2007–2010–2014–2015–2016, whose prevalent trend was clearly on the rise. Outbreak sequences in Chaozhou in 2008 individually formed a clade between Lineages II and III. Interestingly, concerning the recent severe DENV epidemic, since 2006 or 2007 (the previous circulation), Lineages II–IV had not spread continuously, but instead had circulated locally and consecutively every year after several years. It is unclear whether the subsequent lineages originated from strains in 2006 or 2007, or were imported from neighboring countries in that year. In addition, genotype IV was detected only in 2007 and 2010, whereas genotype V circulated from 2009 to 2011, and then, after a 2-year silence, reappeared in 2014 (Figure 4).

**Figure 5.**
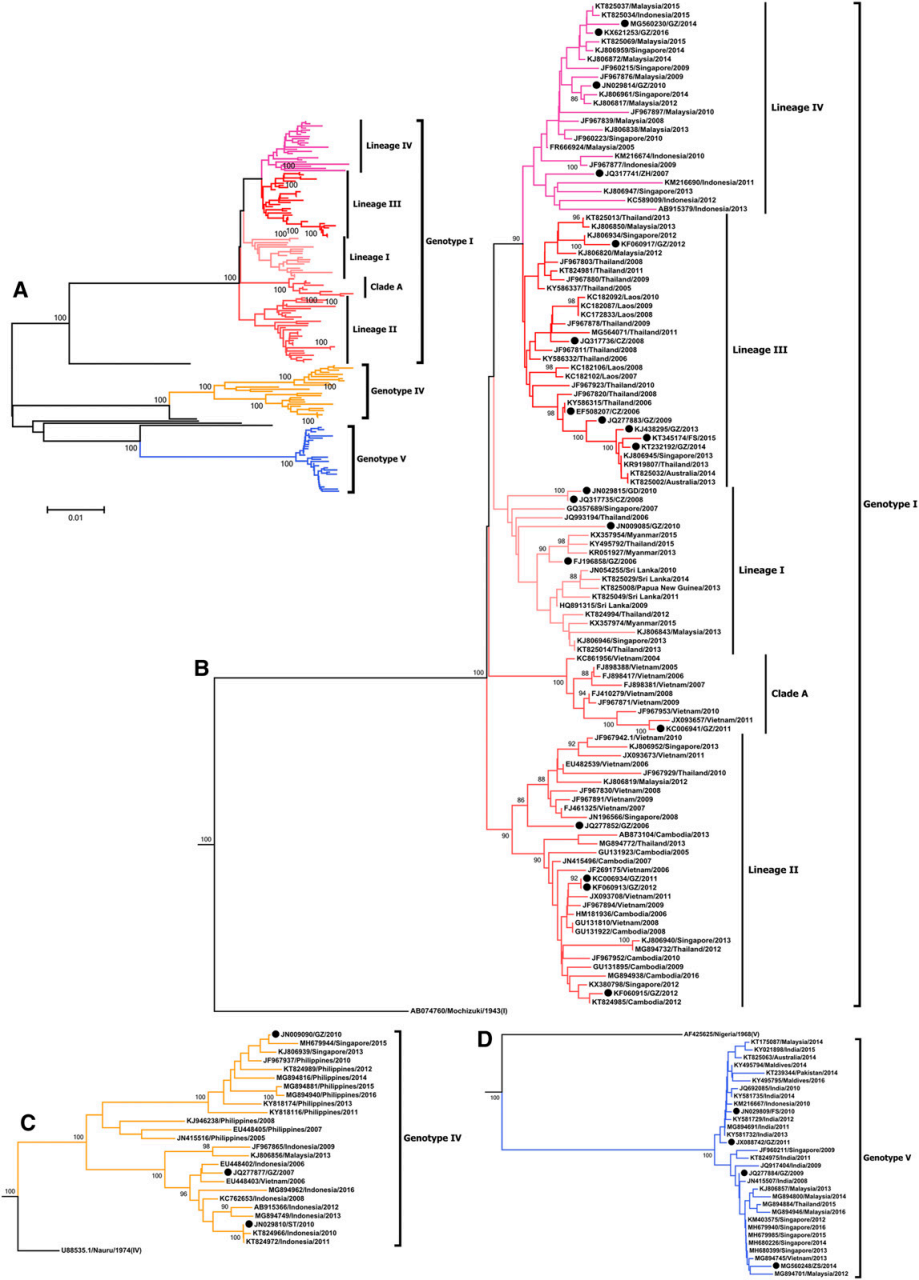
Phylogenetic tree of a molecular evolution database constructed by envelope (E) gene in other countries to identify imported sources in Guangdong since 2005. (**A**–**D**) The same color in genotype I represents the viral sequences that clustered into the same lineage. Yellow and blue represent genotypes IV and V, respectively. (**A**) A similar lineage characterization was revealed when 27 representative sequences (black circles) from each lineage of Guangdong clustered together with 143 sequences from other countries. (**B**–**D**) Phylogenetic tree by E gene sequences of genotypes I, IV, and V, respectively. This figure appears in color at www.ajtmh.org.

We endeavored to clarify the relationship between Guangdong and other countries concerning DENV outbreaks. For this, we illustrated the origin of molecular evolution lineages and the potential of in situ evolution. Representative strains from each lineage were added to the imported database for phylogenetic evolution analysis. In our collection, the molecular evolutionary lineage of the imported database proved to be highly consistent with the local database. The only distinctions were that the evolution time of Lineages I and II changed in genotype I, and DENV1 in Guangzhou, 2011, formed a clade alone, named clade A (Figure 5A). Genotype I, which has extensively circulated in Guangdong and has been detected in each year of the epidemics, displayed some differences with epidemic countries surrounding China. After 2010, Lineage I was not found in Guangdong. Lineage I was very homologous with lineages in Singapore, Thailand, and Sri Lanka in the same epidemic years. However, Lineage I spread in neighboring countries, such as Malaysia, Myanmar, and New Guinea, which contributed to cyclic epidemics (Figure 5B). Lineage II in Guangdong presented a more complicated epidemic situation in countries around China: for example, Lineage II extensively circulated in Vietnam, Singapore, Thailand, Malaysia, and Cambodia, with Vietnam and Cambodia being hot spots. Clade A only formed a cluster with DENV1 in Vietnam (Figure 5B). Recently, Lineage III in Guangdong showed a complicated epidemic situation in surrounding countries as well. Its sequence was related to sequences in Thailand, Laos, Singapore, Malaysia, and elsewhere. In addition, it was related to the co-epidemic in Australia that occurred in 2013 and 2014 (Figure 5B). There were few countries with Lineage IV whose epidemic originated from the sequences in 2007. Only Malaysia, Singapore, and especially Indonesia shared clustering in DENV1 with long-term cyclic epidemics (Figure 5B). In addition, the occurrence of genotypes IV and V was inconsistent in different years. Genotype IV sequences in 2007 shared extensive homology with those in Indonesia, whereasthe strains in 2010 were related to those in the Philippines with long-term cyclic epidemics (Figure 5C). Genotype V was related to the strains in India and Maldives and was divided into clade 2009 and 2014 and clade 2010 and 2011; the sequences shared long-term co-epidemics with India and Singapore, respectively (Figure 5D).

### Protein conformational changes caused by mutation of E protein–specific AAs between DENV1 genotypes I and V.

**Figure 6.**
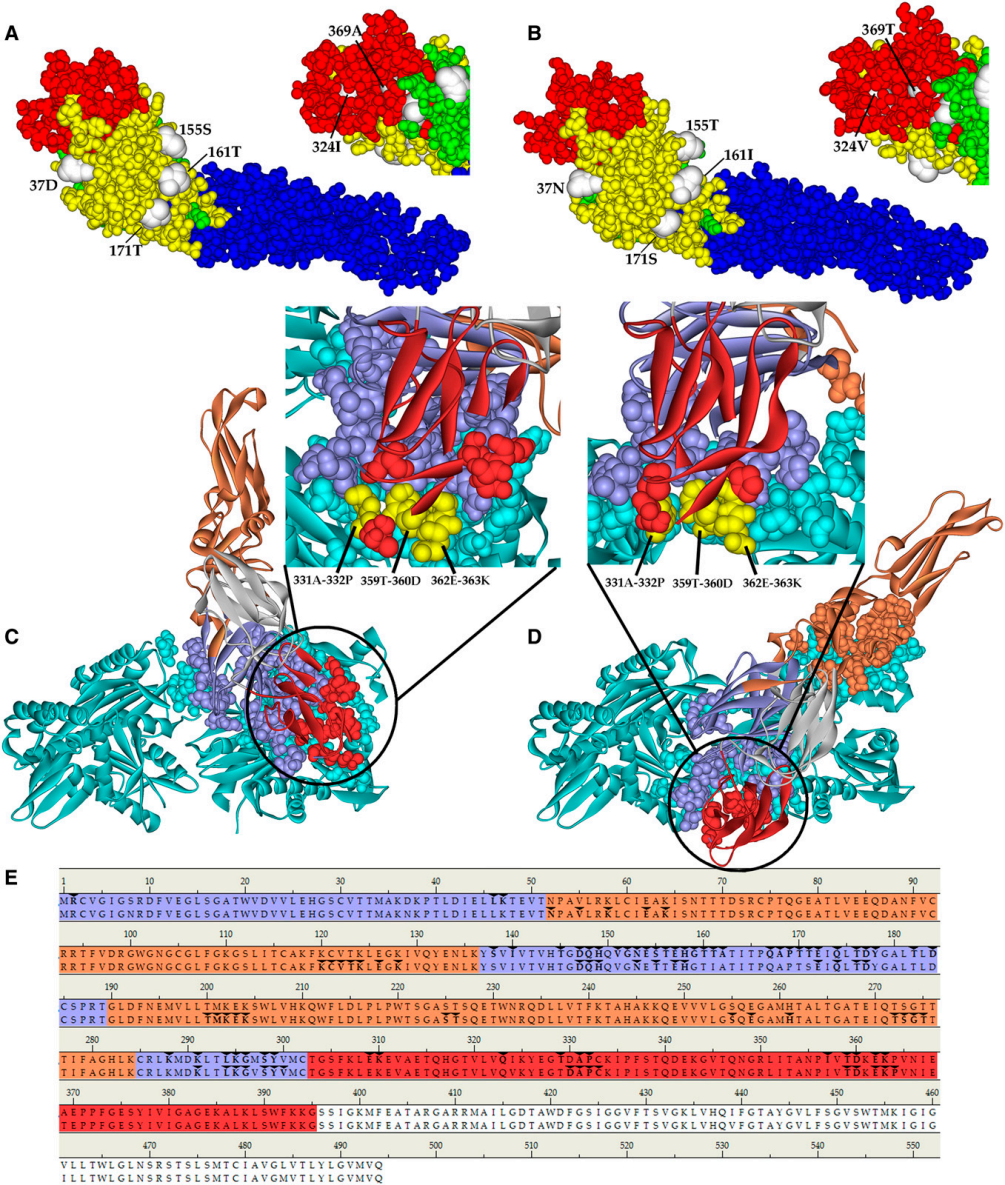
Three-dimensional structure prediction of protein envelope (E) of genotypes I and V and molecular docking with GRP78 protein. (**A**–**B**) Three-dimensional structure of the E protein of genotypes I and V, respectively. Yellow circle: Domain I, blue circle: Domain II, red circle: Domain III, and white circle: substitutions. (**C**–**E**) Docking model and binding interface of the E protein of genotypes I and V with GRP78 protein, respectively. Circles, lines, and backgrounds: Domain I (purple), Domain II (brown), Domain III (red), transmembrane (white), GRP78 (blue), and three identical docking sites (yellow). (E) The aforementioned sequence: genotype I (P1278), the following sequence: genotype V (P1253). This figure appears in color at www.ajtmh.org.

A total of eight uniform AA substitutions in the ectodomain of the E protein were mainly concentrated in domains I (five substitutions) (DI) and III (two substitutions) (DIII) between genotypes I and V, whereas domain II (DII) has only one. Among them, two substitutions caused the protein’s secondary structure to change from β-sheet to coil, E114 (I → L) in DII and E171 (T → S) in DI, whereas only E37 (D → N), E155 (S → T), E161 (T → I), and E171 (T → S) of DI were observed on the 3D conformation surface of the protein (Figure 6A and B, Table 3). Meanwhile, E161 (T → I) and E171 (T → S) of DI were also found in the binding interface between the E protein of genotypes I and GRP78, and other substitutions were not observed in either serotypes (Figure 6E). In the docking model with the GRP78 protein, all three domains of genotype I were involved, whereas genotype V had only DI and DIII. We found three identical docking sites of DIII in the binding interface of the two genotypes: 331A-332P, 359T-360D, and 362E-363K (Figure 6C and D).

**Table 3:** Description of the differences in secondary and three-dimensional (3D) structure between genotypes I and V of DENV1 in Guangdong

Sl. No.	AA position (protein)	Domain (protein E)	Surface in 3D (protein)	P1278 (genotype I)		P1253 (genotype V)	
				AA	Secondary structure	AA	Secondary structure
1	8	I	No	S	Coil	N	Coil
2	37	I	Yes	D	Coil	N	Coil
3	114	II	No	I	β-sheet	L	Coil
4	155	I	Yes	S	Coil	T	Coil
5	161	I	Yes	T	β-sheet	I	β-sheet
6	171	I	Yes	T	β-sheet	S	Coil
7	324	III	No	I	β-sheet	V	β-sheet
8	369	III	No	A	β-sheet	T	β-sheet
9	439	Transmembrane	No	I	β-sheet	V	β-sheet
10	461	Transmembrane	No	V	β-sheet	T	β-sheet
11	484	Transmembrane	Yes	L	β-sheet	M	β-sheet

AA = amino acid; E = envelope; 3D = three-dimensional. The AA positions are in respect to NCBI reference sequence KM204119.1.

## DISCUSSION

The distribution characteristic of DENV1 in Guangdong was determined from its long-term epidemic history. Dengue virus 1 was first detected in Zhongshan, Guangdong Province. Since then, DENV1 epidemics have occurred sporadically in specific regions over 2–3 years.^32^ Until 2006, DENV1 was the leading serotype, which triggered massive outbreaks in Guangzhou.^33^ Thereafter, DENV was circulated continuously across multiple geographies in Guangdong, and the isolated strains branched into several stable molecular evolutionary clades.^34,35^ Since 2009, it has spread to different provinces of Guangzhou, and imported cases were no longer primary. At present, imported and local cases are equally responsible for the DENV outbreaks, which have occurred with a complex pattern wherein all four serotypes have emerged, with DENV1 being the primary. Meizhou was the only city in Guangdong Province in which no cases of DENV were detected since 2005, indicating the criticality of the occurrence of the DENV epidemic in this region.^36,37^ Analyses of the epidemiological trends suggest that the severity of DENV outbreaks has increased since 2012. The rising number of cases and extension of the pandemic to widely distributed areas characterized the large outbreak in 2014, which marked the top of the periodical DENV infections and transmission. With an increase in the number of cases reported between 2016 and 2018, it can be speculated that, in 2019, the severity of the epidemic might increase and require our attention.

The year 2014 witnessed the largest historical outbreak of dengue with the maximum number of cases and the widest distribution. Of 45,189 reported cases, 45,131 indigenous ones accounted for 99.8%, reaching the trend that local ones were dominant in recent years.^38–40^ Previous studies indicated that although three DENV serotypes (DENV1, DENV2, and DENV3) were identified, DENV1 was the major causative agent of this outbreak which had circulated continuously in multiple geographies.^41,42^ So far, the results mentioned were consistent with those of our study. The term “lineage” has been used to denote the viruses clustered in clades at a taxonomic level beneath the genotype. The appearance, change, and reappearance of specific lineages are closely linked to the transmission of those viruses.^43–45^ Here, 56 strains of viruses differing in their E genes were obtained, of which nine and 47 strains belonged to genotypes I and V, respectively. Unlike the strains of genotype I which were linked to two lineages, each strain in genotype V branched into the same clade, forming a stable lineage. However, several lineages from different origins were responsible for the DENV outbreak in 2014. Moreover, strains belonging to genotype V were detected in six areas evaluated in this study. All 13 strains from Shanwei, Chaozhou, and Huizhou gathered together also belonged to this genotype, illustrating that the same lineage was prevalent in multiple regions. Coincidentally, some epidemiological data indicate that DENV1 was restricted only to the local epidemics since 1997,^41^ a phenomenon which was reported by Guo et al.^24^ Overall, the aforementioned evidence strongly opposes the conventional belief that DF in Guangdong was completely imported.

Lee et al. used the term “in situ evolution” to characterize DENV in Singapore through phylogenetic analysis. Results of the analysis explained the correlation between its genetic and evolutionary aspects, suggesting that DENV had lurked locally and reappeared after some time.^46^ Rajarethinam et al.^47^ described that the dengue in Singapore from 2004 to 2016 demonstrated cyclic epidemic patterns dominated by serotypes 1 and 2. However, this in situ evolution, demonstrated by the step-ladder pattern of branching within each clade over time, has not been observed in Guangdong since 2005. Another possible transmission and evolution pattern of DENV broke out in parts (switch of the lineage), followed by silence (change of the lineage), and was then prevalent on a large scale (continuation of the lineage), achieving continuous evolution of a new lineage and the silent circulation of old lineages. In 2006, when DENV1 first became the leading serotype, it was due to a “switch” from the lineages in genotype I, which branched into Lineages I–III at the beginning.^48,49^ From 2007 to 2012, it was observed that the prevalence of DENV was low and it frequently interchanged between the three lineages, leading to the occurrence of epidemics caused by DENV1 belonging to each lineage.^50,51^ However, since 2012, the severity of dengue is increasing because of a continued epidemic caused by Lineage III.^52^ In 2007, a switch in Lineage IV, which was similar to the evolutionary process of Lineage III, was observed. The lineage of genotype IV was first formed in 2007, which reappeared in 2010, and was never detected in recent years.^53,54^ It is reported that genotype V had circulated continuously from 2009 to 2011, followed by an intermittent silence and further reappearance in 2014. This type of molecular evolution was similar to the silent circulation prevalent in Indonesia, Brazil, etc.,^11,55,56^ indicating that the same might happen in Guangdong. Castonguay-Vanier et al.^57^ also described the active circulation of DENV in Laos and the concurrent multiple introductions of new strains from neighboring countries, whereas Moore et al.^58^ claimed that the co-circulation of DENV1–4 in PNG provides molecular evidence of its endemic transmission. Meanwhile, compared with DENV2, DENV3, and DENV4, DENV1, which is the leading serotype in Guangdong, rarely caused severe DF.^59–61^ In addition, because Guangdong is the most densely populated province in southern China, which is surrounded by a large number of dengue-endemic countries and has a subtropical climate providing optimal environmental, social, and biological conditions for mosquito breeding and reproduction, a detailed evaluation of the current dengue epidemic is significant. Nevertheless, there is still a lack of evidence on the local mosquito vector regarding its harboring of DENV during epidemic and nonepidemic periods which can support the evidences of its molecular evolution revealed by the phylogenetic analysis.

Three genotypes (I, IV, and V) in DENV1 from a variety of origins were identified. Data obtained from the characterization of local or imported viruses with similarity in lineages found in Guangdong demonstrated that these viruses, which were identified based on their molecular evolutionary analysis, were also present in many other countries, particularly those neighboring China. Continuous occurrences of the epidemic in Indonesia, Malaysia, and Singapore were consistent with the outbreaks in our country. The epidemiological data demonstrated that the early imported cases in Guangdong had high correlation with those found in the neighboring countries, which indicated that these countries might be the source for the migration of DENV1 to Guangdong.^62,63^ However, specific switches in different genotypes were observed, which are as follows: Myanmar, Thailand, Vietnam, and Laos for genotype I; the Philippines for genotype IV; and India and Maldives for genotype V. The outbreaks of severe epidemics over the years in the neighboring countries and frequent mobility of their populations to China may be able to explain the pandemic pattern of the spread of DENV, which began with its spread to Guangdong Province and then broke out in other parts of the country. A similar conclusion was presented in the study conducted by Sun et al.^64^ regarding the epidemiological characteristics and genetic diversity of DENV in Guangdong in 2014. Nevertheless, since 2012, with an extremely high incidence of cases, DENV spread more rapidly and affected a wider range of populations. The co-epidemic between other countries and Guangdong and the dissemination of different genotypes and serotypes in multiple regions might not align totally with the theory that only the imported cases caused the epidemic. Lee et al.^46^ reported a theory which stated that multiple factors are involved in addition to the ones described in in situ evolution that can explain this phenomenon. However, we still need more evidence to prove the applicability of this hypothesis to the epidemic outbreaks in Guangdong Province.

A prediction of the algorithm of the secondary structure of proteins suggests that it is closely related to the distribution of protein epitopes. The high chemical bond energy of the α-helix and β-sheet enables folding of the protein, making it difficult to bind to the antibody, whereas the β-turn and coil, because of their loose structure, are easily displayed on the surface as antigenic epitopes, facilitating binding of the antibody.^65–67^ Only two substitutions causing changes in the secondary structure from the β-sheet to coil were observed in our study, suggesting that its antigenicity was enhanced. However, E171 (T → S) located on the surface is more likely to be associated with protein function than E114 (I → L), which was located inside the protein. Although DI, the structure found in the central region of the E protein, was not significantly related to the protein function, in our study, majority of the AA substitutions were observed (5/8), and most of them were located on the surface of its 3D structure. Even E161 (T → I) and E171 (T → S) were found in the binding interface between genotype I and GRP78 but not genotype V, further suggesting that the substitution of E171 (T → S) may be playing a key role in the binding of the E protein of genotypes I and V to the receptor. Moraes et al.^68^ found that, with changes in the pH, the specific interaction between DI and DIII of the DENV E protein is destroyed, resulting in its conformational change during entry into the cell, whereas Nayak et al.^69^ also observed the presence of a bundle structure consisting of four polar AA residues at the interface between DI and DIII, of which HIS-282 and HIS317 were unique to DENV1, implying that the change of DI conformation will also affect the realization of DIII function. Domain III has an immunoglobulin-like structure and a functional region where DENV binds to a cellular receptor. Drumond et al.^70^ predicted the structure of the DENV1 E protein in Brazil and observed that the substitution of E306 (S → F) can reduce its interaction with several residues (SER305, LYS307, LYS325, VAL380, VAL324, ILE335, TYR326, and GLY381), resulting in a change in the folding of the area, whereas genotypes I and V of DENV1 in Guangdong have a unified mutation in E324 (I → V). The residue QHG at position E314–E316 of the E protein of DENV2 and 3 is highly conserved, and docking by the ZDOCK method showed that it possibly interacts with the membrane receptor protein TIM-1 ^71^, although we found the same region conserved at E316-E318. Meanwhile, three conserved regions (331A–332P, 359T–360D, and 362E–363K) were concurrently marked in the binding interface of the two genotypes found in Guangdong. Chen et al.^72^ localized the neutralizing determinants of the inhibitory mAbs demonstrating strong effects to a sequence-unique epitope on DIII of the DENV1 E protein, centered near residues T346 and D360 (346TQNGRLITANPIVTD360) which were highly conserved among different genotypes of DENV1 but different from those of the DENV2, DENV3, and DENV4 serotypes and other flaviviruses.

## CONCLUSION

Currently, we believe that vaccination and vector control are the fundamental measures to control DF. However, research on vaccines and mosquito-control measures have not made significant breakthroughs. We need additional information to understand the epidemic situation of DF and evolution of its virus in Guangdong to develop effective preventive and control measures. Epidemiological analysis reveals information on the cities and months during which DF was substantially prevalent and helps to develop mosquito-surveillance and killing strategies. The phylogenetic tree revealed that DENV1, which is the main serotype of the virus, has been prevalent in Guangdong since a long time. The strains isolated from epidemic cases occurring during the same period are homologous, and genotype I has formed a stable evolutionary lineage in recent years. These results suggested that DENV1 may be lurking and circulating in Guangdong, although it cannot be stated with certainty. However, it is highly recommended that we detect the DENV in local mosquito vectors urgently. At the same time, the phylogenetic tree of the input source suggests the possible countries and regions from which importation of DF in Guangdong can occur. This information is of great significance for the development of a plan to monitor the departure and entry of populations from regions with a high incidence of dengue.

## References

[1] Simmons CP Farrar JJ Chau VVN Wills B, 2012 Dengue. New Engl J Med 366: 1423–1432. 22494122 10.1056/NEJMra1110265

[2] Guhasapir D Schimmer B, 2005 Dengue fever: new paradigms for a changing epidemiology. Emerg Themes Epidemiol 2: 1. 15743532 10.1186/1742-7622-2-1 PMC555563

[3] Peng HJ 2012 A local outbreak of dengue caused by an imported case in Dongguan China. BMC Public Health 12: 83. 22276682 10.1186/1471-2458-12-83 PMC3311058

[4] Nogueira RMR Araújo JMGD Schatzmayr HG, 2007 Dengue viruses in Brazil, 1986–2006. Rev Panam Salud Pública 22: 358–363. 18198045 10.1590/s1020-49892007001000009

[5] WHO, 2009 Dengue: Guidelines for Diagnosis, Treatment, Prevention and Control, New ed., Vol. 6. Geneva, Switzerland: World Health Organization, 990. 23762963

[6] World Health Organization, 2019 Dengue and Severe Dengue. Available at: http://www.who.int/mediacentre/factsheets/fs117/en/.

[7] Brady OJ Gething PW Bhatt S Messina JP Brownstein JS Hoen AG Moyes CL Farlow AW Scott TW Hay SI, 2012 Refining the global spatial limits of dengue virus transmission by evidence-based consensus. PLoS Negl Trop Dis 6: e1760. 22880140 10.1371/journal.pntd.0001760 PMC3413714

[8] Bhatt S 2013 The global distribution and burden of dengue. Nature 496: 504–507. 23563266 10.1038/nature12060 PMC3651993

[9] Chambers TJ Chang SH Galler R Rice CM, 1990 Flavivirus genome organization, expression, and replication. Annu Rev Microbiol 44: 649–688. 2174669 10.1146/annurev.mi.44.100190.003245

[10] Holmes EC Twiddy SS, 2003 The origin, emergence and evolutionary genetics of dengue virus. Infect Genet Evol 3: 19–28. 12797969 10.1016/s1567-1348(03)00004-2

[11] De BF Nogueira RM Faria NR Simões JB Nunes PC de Filippis AM dos Santos FB, 2015 Insights of the genetic diversity of DENV-1 detected in Brazil in 25 years: analysis of the envelope domain III allows lineages characterization. Infect Genet Evol 34: 126–136. 26160541 10.1016/j.meegid.2015.07.007

[12] Rico-Hesse R, 2003 Microevolution and virulence of dengue viruses. Adv Virus Res 59: 315–341. 14696333 10.1016/s0065-3527(03)59009-1 PMC3045824

[13] Penny D 1994 The role of models in reconstructing evolutionary trees. Syst Assoc Spec 52: 211.

[14] Lanciotti RS Gubler DJ Trent DW, 1997 Molecular evolution and phylogeny of dengue-4 viruses. J Gen Virol 78: 2279–2286. 9292015 10.1099/0022-1317-78-9-2279

[15] Jackowiak P Kuls K Budzko L Mania A Figlerowicz M Figlerowicz M, 2014 Phylogeny and molecular evolution of the hepatitis C virus. Infect Genet Evol 21: 67–72. 24200590 10.1016/j.meegid.2013.10.021

[16] Xu J Zhong HA Madrahimov A Helikar T Lu G, 2014 Molecular phylogeny and evolutionary dynamics of influenza A nonstructural (NS) gene. Infect Genet Evol 22: 192–200. 24161299 10.1016/j.meegid.2013.10.011

[17] Morenoaltamirano MM Sánchezgarcía FJ Muñoz ML, 2002 Non Fc receptor-mediated infection of human macrophages by dengue virus serotype 2. J Gen Virol 83: 1123–1130. 11961267 10.1099/0022-1317-83-5-1123

[18] Gualano RC Pryor MJ Cauchi MR Wright PJ Davidson AD, 1998 Identification of a major determinant of mouse neurovirulence of dengue virus type 2 using stably cloned genomic-length cDNA. J Gen Virol 79: 437–446. 9519821 10.1099/0022-1317-79-3-437

[19] Klungthong C Putnak R Mammen MP Li T Zhang C, 2008 Molecular genotyping of dengue viruses by phylogenetic analysis of the sequences of individual genes. J Virol Methods 154: 175–181. 18778736 10.1016/j.jviromet.2008.07.021

[20] Wu JY Lun ZR James AA Chen XG, 2010 Dengue fever in mainland China. Am J Trop Med Hyg 83: 664–671. 20810836 10.4269/ajtmh.2010.09-0755 PMC2929067

[21] Liu C Liu Q Lin H Xin B Nie J, 2014 Spatial analysis of dengue fever in Guangdong province, China, 2001–2006. Asia Pac J Public Health 26:58–66. 23343642 10.1177/1010539512472356

[22] He JF Luo HM Liang WJ Zheng K Kang M Liu LP, 2007 Epidemic situation of dengue fever in Guangdong province, China, 1990–2005. Dengue Bull 31: 1–9.

[23] Wu W 2011 Molecular epidemiology of dengue viruses in southern China from 1978 to 2006. Virol J 8: 1–9. 21703015 10.1186/1743-422X-8-322 PMC3138434

[24] Guo R 2014 The prevalence and endemic nature of dengue infections in Guangdong, south China: an epidemiological, serological, and etiological study from 2005–2011. PLoS One 9: e85596. 24465613 10.1371/journal.pone.0085596 PMC3900419

[25] Xiao JP 2016 Characterizing a large outbreak of dengue fever in Guangdong province, China. Infect Dis Poverty 5: 44. 27142081 10.1186/s40249-016-0131-z PMC4853873

[26] Huang L 2016 Epidemiology and characteristics of the dengue outbreak in Guangdong, southern China, in 2014. Eur J Clin Microbiol Infect Dis 35: 269–277. 26700953 10.1007/s10096-015-2540-5

[27] Burland TG, 2000 DNASTAR’s Lasergene sequence analysis software. Methods Mol Biol 132: 71–91. 10547832 10.1385/1-59259-192-2:71

[28] Altschul SF, 1990 Basic local alignment search tool (BLAST). J Mol Biol 215: 403–410. 2231712 10.1016/S0022-2836(05)80360-2

[29] Hall TA, 1999 BioEdit: a user-friendly biological sequence alignment editor and analysis program for Windows 95/98/NT. Nucleic Acids Symp Ser 41: 95–98.

[30] Kumar S Tamura K Nei M, 1994 MEGA: molecular evolutionary genetics analysis software for microcomputers. Comput Appl Biosci 10: 189–191. 8019868 10.1093/bioinformatics/10.2.189

[31] Prasanth GK Divya LM Sadasivan C, 2010 Bisphenol-A can bind to human glucocorticoid receptor as an agonist: an in silicostudy. J Appl Toxicol 30: 769–774. 20669259 10.1002/jat.1570

[32] Luo H He J Zheng K Li L Jiang L, 2002 Analysis on the epidemiologic features of dengue fever in Guangdong province, 1990–2000. Chin J Epidemiol 23: 427–430. 12667352

[33] Liang WJ 2007 Epidemiologlcal analysis of dengue fever in Guangdong province, 2001–2006. South China J Prev Med 33:4–7.

[34] Chen S, 2011 The origin of dengue viruses caused the DF outbreak in Guangdong province, China, in 2006. Infect Genet Evol 11: 1183–1187. 21473933 10.1016/j.meegid.2011.03.025

[35] Zheng K Zhou HQ Yan J Ke CW Maeda A Maeda J Takashima I Kurane I Ma H Xie XM, 2009 Molecular characterization of the, E gene of dengue virus type 1 isolated in Guangdong province, China, in 2006. Epidemiol Infect 137: 73–78. 18387217 10.1017/S0950268808000617

[36] Luo L Liang HY Hu YS Liu WJ Wang YL Jing QL Zheng XL Yang ZC, 2012 Epidemiological, virological, and entomological characteristics of dengue from 1978 to 2009 in Guangzhou, China. J Vector Ecol 37: 230–240. 22548558 10.1111/j.1948-7134.2012.00221.x

[37] Runge-Ranzinger S Mccall PJ Kroeger A Horstick O, 2014 Dengue disease surveillance: an updated systematic literature review. Trop Med Int Health 19: 1116–1160. 24889501 10.1111/tmi.12333 PMC4253126

[38] Huang L 2016 Epidemiology and characteristics of the dengue outbreak in Guangdong, southern China, in 2014. Eur J Clin Microbiol Infect Dis 35: 269–277. 26700953 10.1007/s10096-015-2540-5

[39] Pei W 2016 Molecular characterization and phylogenetic analysis of dengue virus type 1 in Guangdong in 2014. Springerplus 5: 1942. 27933232 10.1186/s40064-016-3604-4 PMC5102991

[40] Li MT Sun GQ Yakob L Zhu HP Jin Z Zhang WY, 2016 The driving force for 2014 dengue outbreak in Guangdong, China. PLoS One 11: e0166211. 27861514 10.1371/journal.pone.0166211 PMC5115708

[41] Shen SQ 2015 Multiple sources of infection and potential endemic characteristics of the large outbreak of dengue in Guangdong in 2014. Sci Rep 5: 16913. 26593240 10.1038/srep16913 PMC4655357

[42] Geng L 2017 Molecular epidemiology demonstrates that imported and local strains circulated during the 2014 dengue outbreak in Guangzhou, China. Virol Sin 32: 63–72. 28120220 10.1007/s12250-016-3872-8 PMC6702253

[43] Mendez JA Usmeciro JA Domingo C Rey GJ Sanchez JA Tenorio A Gallego-Gomez JC, 2010 Phylogenetic history demonstrates two different lineages of dengue type 1 virus in Colombia. Virol J 7: 226. 20836894 10.1186/1743-422X-7-226 PMC2944171

[44] Drumond BP Mondini A Schmidt DJ Bosch I Nogueira ML, 2012 Population dynamics of DENV-1 genotype V in Brazil is characterized by co-circulation and strain/lineage replacement. Arch Virol 157: 2061–2073. 22777179 10.1007/s00705-012-1393-9

[45] Cunha MD Guimarães VN Souza M de Paula Cardoso Dd de Almeida TN de Oliveira TS Fiaccadori FS, 2016 Phylodynamics of DENV-1 reveals the spatiotemporal co-circulation of two distinct lineages in 2013 and multiple introductions of dengue virus in Goiás, Brazil. Infect Genet Evol 43: 130–134. 27223633 10.1016/j.meegid.2016.05.021

[46] Lee KS Lo S Tan SS Chua R Tan LK Xu H Ng LC, 2012 Dengue virus surveillance in Singapore reveals high viral diversity through multiple introductions and in situ, evolution. Infect Genet Evol 12: 77–85. 22036707 10.1016/j.meegid.2011.10.012

[47] Rajarethinam J 2018 Dengue in Singapore from 2004 to 2016: cyclical epidemic patterns dominated by serotypes 1 and 2. Am J Trop Med Hyg 99: 204–210. 29848407 10.4269/ajtmh.17-0819 PMC6085773

[48] Zheng K Zhou HQ Yan J Ke CW Maeda A Maeda J Takashima I Kurane I Ma H Xie XM, 2009 Molecular characterization of the E gene of dengue virus type 1 isolated in Guangdong province, China, in 2006. Epidemiol Infect 137: 73–78. 18387217 10.1017/S0950268808000617

[49] Chen S, 2011 The origin of dengue viruses caused the DF outbreak in Guangdong province, China, in 2006. Infect Genet Evol 11: 1183–1187. 21473933 10.1016/j.meegid.2011.03.025

[50] Fan JC Lin HL Wu HX Wang J Yang SR Liu QY 2013 Spatial and temporal distribution characteristics of dengue fever in Guangdong province, China during 2006–2011. Chin J Vector Biol Control 24: 389–391.

[51] Guo R Peng Z Song T He J Zhong H Li L Liang W, 2014 Current infection status and epidemic risk analysis of dengue fever and chikungunya in Guangdong province, from 1990 to 2012. Zhonghua Liu Xing Bing Xue Za Zhi 35: 167–169. 24739557

[52] Feng L 2018 Distribution of dengue virus serum type I in Guangdong, 2013–2016. Mod Prev Med 45: 3857–3961.

[53] Jiufeng S 2018 The epidemiological characteristics and molecular phylogeny of the dengue virus in Guangdong, China, 2015. Sci Rep 8: 9976. 29967414 10.1038/s41598-018-28349-2 PMC6028473

[54] Zhijun B 2018 Evolutionary and phylodynamic analyses of, dengue virus, serotype I in Guangdong province, China, between 1985 and 2015. Virus Res 256: 201–208. 29990510 10.1016/j.virusres.2018.07.005

[55] Fahri S Yohan B Trimarsanto H Sayono S Hadisaputro S Dharmana E Syafruddin D Sasmono RT, 2013 Molecular surveillance of dengue in semarang, Indonesia revealed the circulation of an old genotype of dengue virus serotype-1. PLoS Negl Trop Dis 7: e2354. 23951374 10.1371/journal.pntd.0002354 PMC3738473

[56] Kotaki T Yamanaka A Mulyatno KC Churrotin S Labiqah A Sucipto TH Soegijanto S Kameoka M Konishi E, 2014 Continuous dengue type 1 virus genotype shifts followed by co-circulation, clade shifts and subsequent disappearance in Surabaya, Indonesia, 2008–2013. Infect Genet Evol 28: 48–54. 25219342 10.1016/j.meegid.2014.09.002

[57] Castonguay-Vanier J 2018 Molecular epidemiology of dengue viruses in three provinces of Lao PDR, 2006–2010. PLoS Negl Trop Dis 12: e0006203. 29377886 10.1371/journal.pntd.0006203 PMC5805359

[58] Moore PR Hurk AFVD Mackenzie JS Pyke AT, 2017 Dengue viruses in Papua New Guinea: evidence of endemicity and phylogenetic variation, including the evolution of new genetic lineages. Emerg Microbes Infect 6: e114. 29259329 10.1038/emi.2017.103 PMC5750459

[59] Ocazionez RE Cortés FM Villar LA Gómez SY, 2006 Temporal distribution of dengue virus serotypes in Colombian endemic area and dengue incidence. Re-introduction of dengue-3 associated to mild febrile illness and primary infection. Mem Inst Oswaldo Cruz 101: 725–731. 17160279 10.1590/s0074-02762006000700004

[60] Ocazionez RE Gómez SY Cortés FM, 2015 Dengue hemorrhagic fever serotype and infection pattern in a Colombian endemic area. Rev Salud Pública (Bogota) 9: 262–274. 10.1590/s0124-00642007000200010 17962844

[61] Jones JM 2016 Binational dengue outbreak along the United States-Mexico border - Yuma County, Arizona, and Sonora, Mexico, 2014. MMWR Morb Mortal Wkly Rep 65: 495–499. 27196619 10.15585/mmwr.mm6519a3

[62] Luo L Liang HY Hu YS Liu WJ Wang YL Jing QL Zheng XL Yang ZC, 2012 Epidemiological, virological, and entomological characteristics of dengue from 1978 to 2009 in Guangzhou, China. J Vector Ecol 37: 230–240. 22548558 10.1111/j.1948-7134.2012.00221.x

[63] Shi Y Li S Li X Zheng K Yuan S Huang J, 2016 Epidemiological and molecular characterization of dengue viruses imported into Guangzhou during 2009–2013. Springerplus 5: 1635. 27722053 10.1186/s40064-016-3257-3 PMC5031575

[64] Sun J Wu D Zhou H Zhang H Guan D He X Cai S Ke C Lin J, 2016 The epidemiological characteristics and genetic diversity of dengue virus during the third largest historical outbreak of dengue in Guangdong, China, in 2014. J Infect 72: 80–90. 26546854 10.1016/j.jinf.2015.10.007

[65] Yanbo L, 2003 Prediction of the secondary structure and B cell epitopes for the M protein of SARS coronavirus. Prog Biotechnol 23: 41–45.

[66] Wood MJ Hirst JD, 2005 Protein secondary structure prediction with dihedral angles. Proteins 59: 476–481. 15778963 10.1002/prot.20435

[67] Zhao W Cao H Yang F Xie Q Zhang B Yu J Wu Q, 2017 Structure and function of the non-structural protein of dengue virus and its applications in antiviral therapy. Curr Top Med Chem 17: 1–10. 27572085 10.2174/1568026616666160829155327

[68] Moraes AH Simonelli L Pedotti M Almeida FC Varani L Valente AP, 2016 Antibody binding modulates conformational exchange in domain III of dengue virus E protein. J Virol 90: 1802–1811. 26637461 10.1128/JVI.02314-15 PMC4733983

[69] Nayak V Dessau M Kucera K Anthony K Ledizet M Modis Y 2009 Crystal structure of dengue virus type 1 envelope protein in the postfusion conformation and its implications for membrane fusion. J Food Prot 83: 662–667. 10.1128/JVI.02574-08 PMC2668458 19244332

[70] Drumond BP 2016 Phylogenetic analysis of dengue virus 1 isolated from south Minas Gerais, Brazil. Braz J Microbiol 47: 251–258. 26887252 10.1016/j.bjm.2015.11.016 PMC4827697

[71] Wadood A Mehmood A Khan H Ilyas M Ahmad A Alarjah M Abu-Izneid T, 2017 Epitopes based drug design for dengue virus envelope protein: a computational approach. Comput Biol Chem 71: 152–160. 29096381 10.1016/j.compbiolchem.2017.10.008

[72] Chen WH Chou FP Wang YK Huang SC Cheng CH Wu TK, 2017 Characterization and epitope mapping of dengue virus type 1 specific monoclonal antibodies. Virol J 14: 189. 28969658 10.1186/s12985-017-0856-8 PMC5625772

